# ‘An ounce of prevention is worth a pound of cure’

**DOI:** 10.1007/s12471-023-01839-3

**Published:** 2023-12-12

**Authors:** Ruud F. Spee, Hareld M. Kemps, Tom Vromen

**Affiliations:** 1 Department of Cardiology, Maxima Medical Center, Veldhoven, The Netherlands; 2 Faculty of Industrial Design, Technical University Eindhoven, Eindhoven, The Netherlands

Benjamin Franklin famously advised fire-threatened Philadelphians in 1736 that ‘An ounce of prevention is worth a pound of cure.’ Looking even further back in history, a famous Chinese proverb states that inferior doctors cure the disease, mediocre doctors treat the impending disease and superior doctors prevent the disease. This tells us that prevention was always an important theme in human history. The beginning of a new year is the time for looking back, to evaluate the lessons learned and set good intentions for the coming year. Citing the title of the article by Kupper et al. in this issue: it is time for a little less conversation and more action.

In this special issue we focus on current perspectives and advancements in secondary prevention and cardiac rehabilitation (CR). We think it is important to stress the fact that all contributions are of Dutch origin. This shows the strength and quality in the field of secondary prevention and CR. Dutch researchers are able to set the standard for Europe. In line with this ambition, the working group of cardiovascular prevention and rehabilitation (CPH) in collaboration with the working group Cardiology and Sport have composed a fellowship (in Dutch aandachtsgebied) Preventive Cardiology based on the curriculum of the European Association of Preventive Cardiology (EAPC) [1]. This will consist of three pillars: prevention, cardiac rehabilitation and sports cardiology. In the quickly evolving and complex field of Preventive Cardiology it is important to educate the future leaders in this field.

However, we all have a duty in prevention and it does not have to take a lot of time. A very brief advice (VBA) on lifestyle given by a cardiologist can already make an impact for our patients as stated by IJzerman et al. in this issue [2].

Psychosocial factors often underlie an unhealthy lifestyle. In our daily practice, this is often overlooked and standardised screening is lacking. Kupper et al. provide practical advice and a framework for future studies [3].

As our population in the consultation room will only get older, it is important to evaluate the benefits and harms of secondary prevention in this specific population. Van Trier et al. quantified the effect of implementing different guidelines in patients over 70, expressed as the potential individual benefit in gain of event-free years [4].

The image of most caregivers and patients about CR is still a group of old men cycling in a small gym with a physiotherapist on the side telling them what to do. In contrast, with advancements in medical technology, personalised treatment plans tailored to individual patients’ needs have become more opportune. This approach allows healthcare professionals to optimise medication regimens and lifestyle modifications, leading to better outcomes. In this respect, for both healthcare professionals and patients, it is crucial to receive regular and accurate feedback on lifestyle behaviour. Goevaerts et al. evaluated a comprehensive app, which can help caregivers and patients to achieve personalised lifestyle goals [5].

Furthermore, the integration of remote patient monitoring has revolutionised the delivery of CR. Patients can easily access information and resources from the comfort of their homes, and healthcare providers can remotely monitor crucial health indicators and provide timely guidance and support. This has resulted in enhanced patient engagement and improved adherence to treatment plans. Still, several barriers have to be taken before telerehabilitation is widely implemented in the daily practice of CR. Brouwers et al. provide us guidance to take the next steps [6].

New populations are appearing in CR, such as patients with obesity and/or atrial fibrillation. This topic was studied in the Opticare XL study. Short-term results were positive but in the long term effects did not last. Are we on the right track for these patients? Den Uijl et al. demonstrate which lessons can be learned [7].

In the paper by Van Til et al., [8] we can appreciate the importance of collaboration in CR when it comes to screening for cognitive impairment after cardiac arrest. Barriers are solvable but a few hurdles have to be taken.

Recently, Zorginstituut Nederland stated that CR has insufficient scientific evidence to be prescribed in patients with stable angina pectoris [9], despite a class Ia in national and international guidelines [10]. One should wonder why are we treating patients with stable angina pectoris, peripheral artery disease or a cerebrovascular accident differently with respect to cardiovascular risk management and rehabilitation programs, when the underlying cause is atherosclerosis in the vast majority with the same underlying risk factors. In addition, ‘presumed absence of evidence does not mean evidence of absence’ [11]. Heutinck et al. provide an overview of the physiological mechanisms underlying the potential beneficial effects of exercise-based CR as a first-line treatment for angina pectoris [12]. The ongoing PRO-FIT trial may provide answers for contemporary knowledge gaps in this area [13].

Moreover, in 2024 an update of the 2011 multidisciplinary CR guideline is expected, which will help caregivers in CR to provide care according to the most actual scientific standard.

In summary, secondary prevention and cardiac rehabilitation have evolved significantly. With the advancements in technology and a holistic approach to patient care, these interventions have become more effective and accessible, leading to improved cardiovascular outcomes and better quality of life for individuals recovering from cardiac events.

We wish the reader all the best for 2024, with exciting times in secondary prevention and CR ahead of us. Let’s hope that the upcoming government will listen to their American historical counterpart Franklin and aim high for prevention.
